# CD4/CD8 + T cells, DC subsets, Foxp3, and IDO expression are predictive indictors of gastric cancer prognosis

**DOI:** 10.1002/cam4.2596

**Published:** 2019-10-20

**Authors:** Fangxuan Li, Yao Sun, Jinchao Huang, Wengui Xu, Juntian Liu, Zhiyong Yuan

**Affiliations:** ^1^ Department of Radiotherapy Tianjin's Clinical Research Center for Cancer Key Laboratory of Cancer Prevention and Therapy Tianjin Medical University Cancer Institute and Hospital National Clinical Research Center for Cancer Tianjin China; ^2^ Department of Cancer Prevention Tianjin Medical University Cancer Institute and Hospital Tianjin China; ^3^ Department of Molecular Imaging and Nuclear Medicine Tianjin Medical University Cancer Institute and Hospital Tianjin China

**Keywords:** dendritic cell, gastric cancer, indoleamine 2,3‐dioxygenase, regulator T cell

## Abstract

**Background:**

The tumor microenvironment represents an abnormal niche containing numerous factors, such as T cells, dendritic cells (DCs), regulatory T cells (Tregs), and indoleamine 2,3‐dioxygenase (IDO), involved in maintaining immune homeostasis and tolerance. All these factors may influence the choice of therapy and the clinical outcomes.

**Methods:**

Flow cytometry was performed to identify CD4+/CD8 + T cells and DCs, and immunohistochemistry was used to evaluate IDO and Forkhead Box P3 (Foxp3) expression; these experiments were performed in order to explore the clinical and prognostic significance of CD4/CD8 + T cells, DCs, Tregs, and IDO expression in gastric carcinoma.

**Results:**

Smaller tumor size was correlated with higher expression levels of peripheral CD4 + T cells (*P* = .003) and CD8 + T cells (*P* = .002), and lower IDO expression (*P* = .044) in tumors. Well‐differentiated gastric carcinomas displayed higher peripheral (*P* = .029) and tumor‐infiltrating CD4 + T cell (*P* = .009) populations and a higher tumor‐infiltrating DC1/DC2 ratio (*P* = .048). Gastric cancer in the early T stages exhibited higher populations of peripheral DC2s (*P* = .044) and a higher tumor‐infiltrating DC1/DC2 ratio (*P* = .012). Gastric cancer at the N0 stage had lower tumor‐infiltrating DC2s (*P* = .032) and a higher DC1/DC2 ratio (*P* = .037). IDO expression was positively correlated with tumor‐infiltrating Foxp3 + Tregs (*P* < .001) as well as DC2s (*P* < .001), whereas it was negatively correlated with the tumor‐infiltrating CD4/CD8 + T cell ratio (*P* = .023). Tumor‐infiltrating Foxp3 + Treg was positively correlated with tumor‐infiltrating DC2s (*r* ^2^ = 0.772; *P* < .001). At T, N, and TNM stages, the expression levels of peripheral DC2s, tumor‐infiltrating DC1/DC2 ratios, Foxp3 + Tregs, and IDO were significantly correlated with prognosis (*P* < .05). The T stage and peripheral DC2s were significant risk factors for overall survival.

**Conclusion:**

Immunocompetent cells and humoral immune factors, including DC2s, CD4+/CD8 + T cells, Foxp3 + Tregs, and IDO, interact with each other to compose a complex community of tumor immune microenvironment, ultimately affecting tumor progression and survival of gastric cancer.

## Graphical abstract

The tumor microenvironment represents an abnormal niche containing numerous factors, such as T cells, DCs, Tregs, and IDO, involved in maintaining immune homeostasis and tolerance. We confirmed that the expression levels of peripheral and tumor‐infiltrating DC2s, CD4+/CD8 + T cells, Foxp3 + Tregs, and IDO were correlated with each other as well as with clinicopathological characters and gastric cancer prognosis.

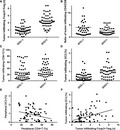

## 1 INTRODUCTION

Conditions within the tumor microenvironment are abnormal. However, malignant cells usually enable and enhance general regulatory functions that maintain homeostasis and immune tolerance in the tumor microenvironment. Lately, many research studies have focused on the prognostic and predictive value of certain immune cell types.^1^ These immune cells comprise innate immune system cells and adaptive immune system cells; the former include dendritic cells (DCs), natural killer (NK) cells, macrophages, and neutrophils among others, while the latter include T and B lymphocytes.^2^

Numerous studies have confirmed that DCs are crucial for tumor immunity in gastric carcinoma.^3^ Many clinical trials have tested DC‐based gastric cancer treatment regimens.^4^ Some DC‐associated inflammatory factors are predictive of gastric carcinoma prognosis.^5^, ^6^ CD83 + DC cells in primary gastric tumors and regional lymph nodes, as well as human lymphocyte antigen (HLA)‐G‐expressing DCs in peripheral blood, are associated with a poor gastric carcinoma prognosis.^7^ Two subtypes of circulating DC precursor populations, DC1 and DC2, have been clearly identified to date. DC1 cells (myeloid DC, mDC), which expresses high levels of CD11c and low levels of CD123, have been identified as key initiators of Th1 T cells, while DC2 cells (plasmacytoid DC, pDC), which express high levels of CD123 antigen, may regulate Th2 T cells.^8^

Regulatory T cells (Tregs) are a subset of CD4 + CD25+T cells. The Forkhead Box P3 (Foxp3) protein is a specific biomarker commonly used to identify Tregs. Tregs inhibit tumor reaction and enable immune tolerance via immune suppression of T cells or secretion of immunosuppressive cytokines, such as IL‐10 and transforming growth factor‐*β* (TGF‐*β*).^9^ Tregs play a fundamental role in maintaining immune tolerance, preventing immune reaction, and lessening immune response.^10^ Treg infiltration of tumor tissues has been extensively evaluated in “immunological character” studies.^11^ These studies found an increase in Treg counts among all tumor‐infiltrating lymphocytes (TILs), particularly in tumors with high immune cell infiltration.^12^ Tregs suppress antitumor immune response, and thereby promote tumor progression. Thus, an increase in Tregs is proposed to be correlated with poor prognosis. Hence, Treg infiltration of tumors has been associated with shorter overall survival (OS) in most solid malignancies.^13^

Indoleamine 2,3‐dioxygenase (IDO) is an important immunosuppressive factor. IDO is constitutively expressed by tumor cells and certain immunosuppressive cells. Numerous studies have confirmed that IDO expression may promote the evasion of cancer cells from immunological surveillance.^14^ This may give tumors a survival advantage.^15^ Increasing expression of IDO exerts two main effects on the tumor microenvironment: depletion of tryptophan (Trp) and accumulation of toxic kynurenine (Kyn) metabolites. Once DCs are stimulated by Toll‐like receptor 9 (TLR‐9) ligation, they upregulate the expression of B‐7 ligands and HLA‐DR antigen, thereby inducing IDO to promote Treg induction.^16^ The IDO effect is an important mechanism by which DC2 induces the generation of Tregs by naive CD4 + T cells.

Globally, gastric carcinoma is the fifth most common malignant disease. In 2012, 952 000 new cases were diagnosed and 720 000 gastric cancer‐related deaths were reported.^17^ Gastric cancer remains a noticeable public health issue, especially in Asian countries such as Japan, China, and Korea. Although, new target therapies based on molecular mechanisms associated with gastric cancer have been developed, prognoses have not improved significantly. Disease risk assessment based on tumor invasiveness, lymph node involvement, distant metastasis (TNM staging), and histological grading is insufficient for predicting the survival of individual gastric cancer patients. Therefore, new prognostic biomarkers are urgently needed. Certain peripheral and tumor‐infiltrating immune cells, such as NK cells, CD3 + T cells, CD8 + T cells, and Foxp3 + Tregs, that are reportedly correlated with better gastric cancer prognoses, have been recommended for predicting prognosis and assessing therapeutic outcomes at the clinical level.^18^, ^19^

A previous study conducted by us in 2013 and 2014,^20^, ^21^ evaluated the association between certain tumor‐infiltrating immune cells, including memory T cells, DC subsets, and IDO expression, with clinicopathological features of gastric cancer. However, an evaluation of prognostic value was not included in that study. In this study, we present an evaluation of the DC subset and CD4/CD8 + T cells in peripheral blood and cancer tissue samples as well as Foxp3 + Tregs and IDO expression in gastric cancer tissue, with the objective of determining whether peripheral and tumor‐infiltrating CD4/CD8 + T cells, DC subsets, and Tregs were correlated with each other and IDO expression as well as correlated with the clinicopathological features of gastric cancer and its prognosis.

## 2 METHODS

### 2.1 Clinical data

Criteria for inclusion of the study were as follows: (a) Gastric cancer patients who received radical resection for stomach tumor from 1 March 2011 to 1 July 2013 at the Tianjin Medical University Cancer Institute and Hospital; and (b) All diagnoses were confirmed as gastric adenocarcinoma via histological examination. All clinical data were analyzed according to the 8th stomach cancer tumor‐node‐metastasis (TNM) staging classification of the Union of International Control Cancer (UICC).

Gastric cancer patients who had contracted immune system diseases or infectious diseases were eliminated. A total of 99 patients, consisting of 63 males and 33 females with a mean age of 59.44 ± 10.92 years, were enrolled in this study. This research project was approved by the Ethics Committee of the Tianjin Cancer Institute and Hospital. Written consent was obtained from each enrolled patient.

### 2.2 Preparation of peripheral blood mononuclear cells

Two milliliter of fasting venous blood was drawn into heparinized tubes. Peripheral blood mononuclear cells (PBMCs) were prepared using density gradient centrifugation on Lymphoprep (Amersham Bioscience) for 25 minutes at 600 *g* under room temperature. Next, PBMCs were washed twice with phosphate‐buffered saline (PBS).

### 2.3 Preparation of cell suspension from cancer and normal tissues

Cancer tissues obtained from the 99 gastric cancer patients were transferred to the laboratory. Necrotic tissues and normal tissues were removed leaving tumor tissues. The tissues were sectioned, suspended in 20 mL of PBS, and incubated for 1.5 hours at 37°C, while shaking intermittently. Next, the tissue samples were subjected to gentle mechanical dispersal using a tissue sieve fitted with a 50 mesh sieve and pestle. The cell suspension was then passed twice through a syringe with a 22‐gauge needle. Cells were placed in DMEM supplemented with FBS media, washed twice, and spun for 7 minutes at 1000 rpm. Cells were then washed again with PBS.

### 2.4 Flow cytometry of CD4+/CD8 + T cells and DC cells

The following antibodies were used for T‐cell surface marker analysis: CD8 FITC, CD3 PerCP, and CD4 PE. For DCs, the following antibodies were used for surface marker analysis: Lin1 FITC, HLA‐DR PerCP, CD11c PE, and CD123 PE (BD Pharmingen). Cells were labeled in TrueCount tubes (BD Pharmingen) using the above antibodies. Isotype‐matched IgG1 was applied and set as a control to decrease nonspecific staining.

Cancerous cells were stained with the following antibodies in situ: CD3/CD4/CD8 and Lin1/HLA‐DR/CD11c/CD123. Cells (1 × 10^8^) were labeled on ice using these antibodies for 30 minutes in the dark. Next, the cells were washed twice with PBS containing 0.2% bovine serum albumin and fixed with 1% paraformaldehyde. Finally, the cells were analyzed using a FACS Aria Flow Cytometry system (Becton Dickinson). In T cells, the ratios of CD3 + CD4+T cells and CD3 + CD8+T cells to T cells (CD3 + T cells), respectively, were evaluated. In DCs, the ratios of DC1 (Lin1‐HLA‐DR + CD11c+) and DC2 (Lin1‐HLA‐DR + CD123+) to HLA‐DR + Lin1‐cells, respectively, were simultaneously evaluated in two separate tubes. At least 50 000 events were counted for each accession. Each sample was analyzed more than three times.

### 2.5 IDO expression, Foxp3 expression, and scoring

#### 2.5.1 Immunohistochemistry

In situ IDO expression and Foxp3 + Treg expression in tumor normal gastric mucosa tissues were examined via immunohistochemical staining. Formalin‐fixed, paraffin‐embedded samples were cut into 4‐μm sections. The sections were dewaxed in xylene and hydrated using an alcohol gradient. Next, samples were blocked using hydrogen peroxide in absolute methanol for 30 minutes. The antigen was heated in a microwave in citrate buffer for 10 minutes. Sections were then allowed to cool down to a normal temperature and blocked with 1% sheep serum. Next, sections were incubated with rabbit polyclonal antibodies against IDO (MilliporeSigma) or mouse monoclonal antibody against human Foxp3 (eBioscience) in a dilution overnight at 4°C, and then incubated with peroxidase‐conjugated AffiniPure goat IgG (Zhongshanjinqiao, Beijing, China). Following this, samples were incubated again with diaminobenzidine tetrahydrochloride (DAB) before hematoxylin staining. PBS was used as a negative control.

#### 2.5.2 IDO expression scoring

IDO expression was assessed semiquantitatively according to IDO‐stained cancer cell percentage and staining intensity. The IDO‐stained cancer cell percentage was scored as follows: 0 (when <5% of cells stained negative); 1 (5%‐25%); 2 (26%‐50%); 3 (51%‐75%); and 4 (>76%). The staining intensity was evaluated as follows: 0 (no staining/negative controls); 1 (weak staining); 2 (moderate staining); and 3 (intense staining). The final score was evaluated by sum indexes of both as follows: (−), (+), (++), and (+++) were indicative of 0‐2, 3‐5, 6‐8, and 9‐12, respectively. Here (−) and (+) were defined as low expression, while (++) and (+++) were defined as high expression.

#### 2.5.3 Scoring of Foxp3 expression

Foxp3 expression was directly evaluated via positive cell staining index. Positive cell staining index = number of positive cells/number of total cells × 100%.

Five different areas were assessed in each patient, and the mean score was set as the final expression score.^22^ Each case was assessed by two pathologists blinded to each other in the absence of clinical data. Where an inconsistency arose, assessment by a third pathologist was obtained to achieve consensus.

### 2.6 Statistical methods

Pearson correlation and Spearman analysis were used to evaluate correlation. Chi‐square and the Fisher's exact tests were applied for categorical variables. The independent *t* test was used to compare continuous variables, and data were expressed as Mean ± SD. Survival times were analyzed and compared using Kaplan‐Meier and Log‐rank tests. COX regression was used to obtain multivariate hazard ratios for prognosis. Statistical significance was set at *P* < .050 (two‐tailed). All analyses were performed using SPSS version 19.0.

## 3 RESULTS

### 3.1 Association between peripheral T cells, DCs, and clinicopathological features

**Figure 1:**
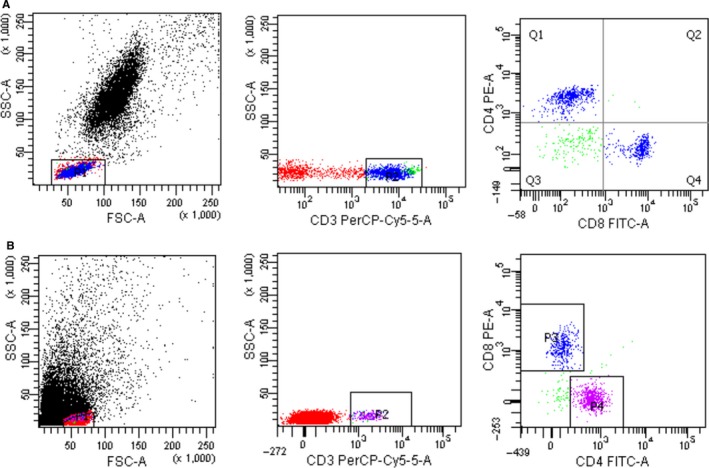
Flow cytometry plots of peripheral and tumor‐infiltrating CD4 + and CD8 + T cells. (A) peripheral blood CD4 + T cells and CD8 + T cells; (B) tumor‐infiltrating CD4 + T cells and CD8 + T cells

**Figure 2:**
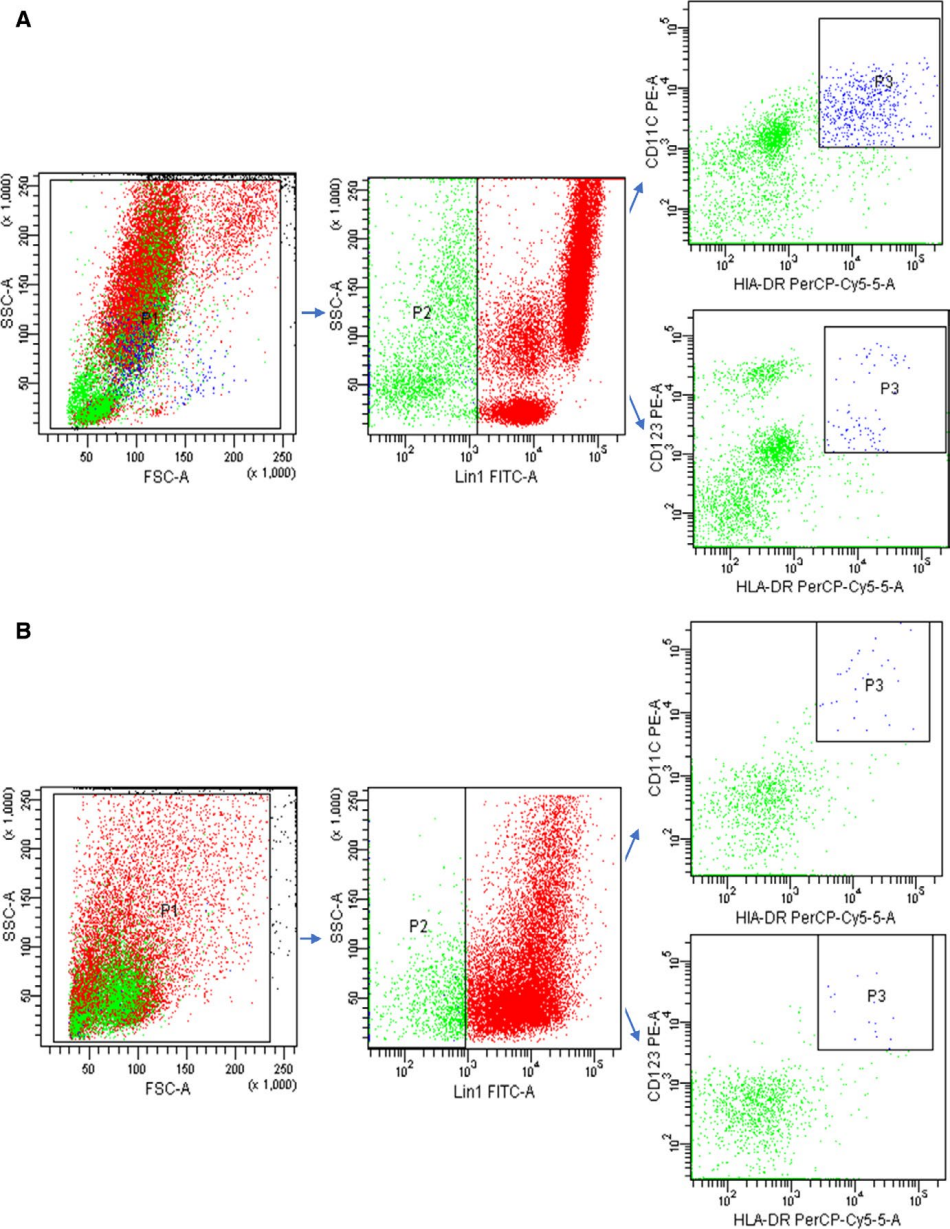
Flow cytometry plots of peripheral and tumor‐infiltrating DC and its subsets. (A) peripheral DC and its subsets; (B) tumor‐infiltrating DC and its subsets

Peripheral and tumor‐infiltrating T cells and DCs were identified via flow cytometry (Figures 1 and 2). Gastric cancer patients with smaller tumor size (<5 cm) exhibited higher levels of CD4 + T cells (*P* = .003) and CD8 + T cells (*P* = .002). Patients with well‐differentiated gastric carcinoma displayed higher levels of CD4 + T cells (*P* = .029). In DC subsets, lower levels of DC2 were seen in more advanced T stage gastric cancer (*P* = .044). All results are shown in Table 1.

**Table 1:** The association between peripheral T cell, DCs, and clinicopathological features of gastric cancer patients

		T cells			DCs		
		CD4	CD8	CD4/CD8	DC1	DC2	*DC1/DC2*
	n	99	99	99	99	99	99
Age							
<50	51	44.47 ± 15.52	29.21 ± 11.03	1.65 ± 0.72	2.59 ± 1.78	0.86 ± 0.70	6.53 ± 8.98
≥50	48	43.19 ± 14.96	27.160 ± 9.11	1.63 ± 0.44	2.304 ± 1.36	0.83 ± 0.69	5.59 ± 6.19
		0.416	1.006	0.187	0.818	0.220	0.585
		0.678	0.317	0.852	0.382	0.826	0.560
Sex							
Male	63	42.62 ± 15.32	28.58 ± 10.48	1.55 ± 0.47	2.35 ± 1.09	0.85 ± 0.72	6.09 ± 8.43
Female	36	45.99 ± 14.91	27.58	1.79 ± 0.749	2.63 ± 1.42	0.84 ± 0.66	6.08 ± 6.66
		1.063	0.473	1.714	0.825	0.035	0.007
		0.290	0.290	0.093	0.412	0.972	0.994
Tumor size							
<5 cm	57	47.74 ± 13.18	30.85 ± 10.24	1.64 ± 0.47	2.31 ± 1.49	0.82 ± 0.64	6.49 ± 9.19
≥5 cm	42	38.56 ± 16.25	24.64 ± 8.94	1.63 ± 0.737	2.69 ± 1.72	0.88 ± 0.78	5.44 ± 4.71
		3.099	3.143	0.044	1.144	0.382	0.638
		0.003	0.002	0.965	0.256	0.703	0.525
Bormann type							
I + II	37	43.31 ± 16.53	25.34 ± 9.76	1.78 ± 0.62	2.36 ± 1.26	0.88 ± 0.79	7.16 ± 9.34
III + IV	62	44.17 ± 14.42	29.93 ± 10.05	1.55 ± 0.57	2.52 ± 1.77	0.82 ± 0.64	5.84 ± 6.61
		0.273	1.945	1.919	0.465	0.386	1.042
		0.786	0.057	0.058	0.643	0.701	0.300
Clinical T stage							
T1, T2	20	40.95 ± 12.47	25.22 ± 11.17	1.77 ± 0.50	2.88 ± 1.60	0.89 ± 0.74	7.38 ± 6.56
T3, T4	79	44.58 ± 15.78	28.98 ± 9.8	1.60 ± 0.62	2.34 ± 1.558	0.65 ± 0.51	5.73 ± 8.05
		0.956	1.486	1.163	1.347	1.721	0.836
		0.342	0.140	0.248	0.181	0.044	0.405
Clinical N stage							
N0	35	40.33 ± 12.61	27.75 ± 12.61	1.59 ± 0.50	2.65 ± 1.51	0.88 ± 0.60	4.81 ± 4.18
N1‐N3	64	45.77 ± 16.19	28.47 ± 8.84	1.67 ± 0.64	2.34 ± 11.63	0.82 ± 0.74	6.78 ± 8.92
		1.722	0.337	0.636	0.886	0.392	1.178
		0.088	0.760	0.526	0.378	0.696	0.242
Clinical stage							
I, II	37	41.03 ± 12.61	28.23 ± 12.13	1.58 ± 0.48	2.61 ± 1.48	0.84 ± 0.61	5.62 ± 5.70
III, IV	62	45.53	28.21 ± 8.86	1.67 ± 0.65	2.36 ± 1.66	0.85 ± 0.74	6.37 ± 8.78
		1.436	0.821	0.758	0.725	0.069	0.446
		0.154	0.414	0.450	0.471	0.945	0.657
Histological grade(‐differentiated)							
Well	28	48.16 ± 13.06	27.81 ± 10.62	1.60 ± 0.62	2.47 ± 1.72	0.864 ± 0.675	5.01 ± 5.05
Poor	71	42.15 ± 15.70	29.26 ± 8.92	1.72 ± 0.52	2.41 ± 1.23	0.79 ± 0.75	8.86 ± 11.41
		2.221	0.642	0.941	0.172	0.427	0.165
		0.029	0.522	0.349	0.712	0.670	0.109

### 3.2 The association between tumor‐infiltrating T cells, DCs, and clinicopathological features

Patients with well‐differentiated gastric cancer had higher levels of tumor‐infiltrating CD4 + T cells (*P* = .009); (Table 2). Gastric cancer at the T1‐T2 stage exhibited a higher tumor‐infiltrating DC1/DC2 ratio than T3‐T4 stage (*P* = .012). Patients with gastric cancer at N0 stage had a lower level of DC2s (*P* = .032) and a higher tumor‐infiltrating DC1/DC2 ratio (*P* = .037). Patients with well‐differentiated gastric carcinoma had a higher tumor‐infiltrating DC1/DC2 ratio (*P* = .048).

**Table 2:** The association between tumor‐infiltrating T cell, DCs, and clinicopathological features of gastric cancer patients

Clinicopathological features		T cells			DCs			*Foxp3 + Treg*	*IDO*	
		CD4	CD8	*CD4/CD8*	DC1	DC2	*DC1/DC2*		*+*	−
	n	99	99	99	99	99	99	99	52(%)	47(%)
Age										
<50	51	66.56 ± 21.92	30.83 ± 22.04	3.34 ± 2.72	0.87 ± 0.83	0.67 ± 0.85	3.96 ± 8.91	3.40 ± 1.46	33(52.4)	30(47.6)
≥50	48	64.79 ± 17.17	30.50 ± 22.77	4.12 ± 4.39	0.86 ± 1.40	0.63 ± 0.67	1.93 ± 1.88	3.03 ± 2.29	19(52.8)	17(47.2)
		0.444	0.073	1.070	0.081	0.299	1.521	0.958	0.001	
		0.658	0.942	0.287	0.936	0.766	0.134	0.334	1.000	
Sex										
Male	63	64.84 ± 21.53	30.03 ± 22.33	4.05 ± 4.29	0.91 ± 0.67	0.63 ± 0.84	3.51 ± 8.33	3.31 ± 2.03	24(47.1)	27(52.9)
Female	32	67.21 ± 16.13	31.78 ± 22.48	3.13 ± 1.873	1.1 ± 1.54	0.69 ± 0.63	2.20 ± 1.81	3.07 ± 1.69	28(58.3)	20(41.7)
		0.575	0.375	1.229	1.691	0.383	0.872	0.606	1.260	
		0.566	0.709	0.222	0.104	0.702	0.386	0.637	0.316	
Tumor size										
<5 cm	57	64.05 ± 19.35	29.72 ± 21.48	3.22 ± 2.08	0.86 ± 1.23	0.66 ± 0.84	3.59 ± 8.48	3.12 ± 1.77	35(61.4)	22(38.6)
≥5 cm	42	67.95 ± 20.14	31.94 ± 23.53	4.38 ± 4.95	0.82 ± 0.92	0.64 ± 0.66	2.14 ± 1.70	3.35 ± 2.09	17(40.5)	25(59.5)
		0.974	0.492	1.590	0.005	0.163	0.982	0.606	4.247	
		0.333	0.624	0.115	0.996	0.871	0.329	0.546	0.044	
Bormann type										
I + II	37	69.92 ± 15.12	35.70 ± 24.69	3.24 ± 2.64	0.56 ± 0.72	0.54 ± 0.82	4.69 ± 10.58	2.92 ± 1.97	23(62.2)	14(37.8)
III + IV	62	63.18 ± 21.68	27.66 ± 20.34	3.99 ± 4.08	1.04 ± 1.27	0.72 ± 0.74	2.06 ± 2.23	3.39 ± 1.86	29(46.8)	33(53.2)
		1.817	1.755	1.008	2.234	1.027	1.783	1.188	2.200	
		0.072	0.082	0.316	0.028	0.307	0.078	0.238	0.152	
Clinical T stage										
T1, T2	20	64.89 ± 14.03	32.16 ± 22.83	1.77 ± 0.50	1.27 ± 1.84	0.45 ± 0.56	6.29 ± 13.09	2.21 ± 1.27	15(75.0)	5(25.0)
T3, T4	79	60.91 ± 20.94	30.29 ± 22.28	1.60 ± 0.62	0.74 ± 0.77	0.71 ± 0.81	2.05 ± 2.30	3.47 ± 1.96	37(46.8)	42(53.2)
		0.206	0.334	1.163	1.239	1.309	2.554	2.737	5.077	
		0.837	0.739	0.248	0.229	0.194	0.012	0.007	0.027	
Clinical N stage										
N0	35	64.99 ± 23.43	30.20 ± 23.40	3.76 ± 3.26	1.02 ± 1.48	0.46 ± 0.49	4.89 ± 10.31	3.07 ± 2.17	23(65.7)	12(34.3)
N1‐N3	64	66.09 ± 17.49	30.92 ± 21.83	3.69 ± 3.82	0.77 ± 0.82	0.78 ± 0.89	1.82 ± 1.83	3.30 ± 1.76	29(45.3)	35(56.5)
		0.266	0.153	0.810	1.013	2.176	2.120	0.581	3.717	
		0.791	0.879	0.936	0.314	0.032	0.037	0.562	0.061	
Clinical stage										
I, II	37	65.61 ± 22.92	29.73 ± 22.83	3.74 ± 3.17	0.97 ± 1.45	0.44 ± 0.48	4.67 ± 8.06	3.01 ± 2.19	25(67.6)	12(34.3)
III, IV	62	65.76 ± 17.67	31.23 ± 22.12	3.70 ± 3.88	0.79 ± 0.83	0.80 ± 0.89	1.86 ± 1.86	3.33 ± 1.78	29(45.3)	35(54.7)
		0.036	0.321	0.061	0.725	0.069	1.959	0.821	3.717	
		0.972	0.749	0.951	0.471	0.945	0.053	0.414	0.061	
Histological grade (‐differentiated)										
Well	28	62.48 ± 20.91	30.32 ± 23.81	3.73 ± 3.75	0.83 ± 1.09	0.69 ± 0.86	3.63 ± 7.94	3.17 ± 1.81	37(52.1)	34(47.9)
Poor	71	73.88 ± 13.24	31.53 ± 18.31	3.68 ± 3.31	0.95 ± 1.20	0.56 ± 0.48	1.59 ± 0.93	3.33 ± 2.16	15(53.6)	13(46.4)
		2.678	0.242	0.052	0.482	0.760	1.989	0.370	0.017	
		0.009	0.809	0.941	0.631	0.449	0.048	0.712	1.000	

### 3.3 The association between tumor‐infiltrating IDO expression and Foxp3 + Treg and clinicopathological features

**Figure 3:**
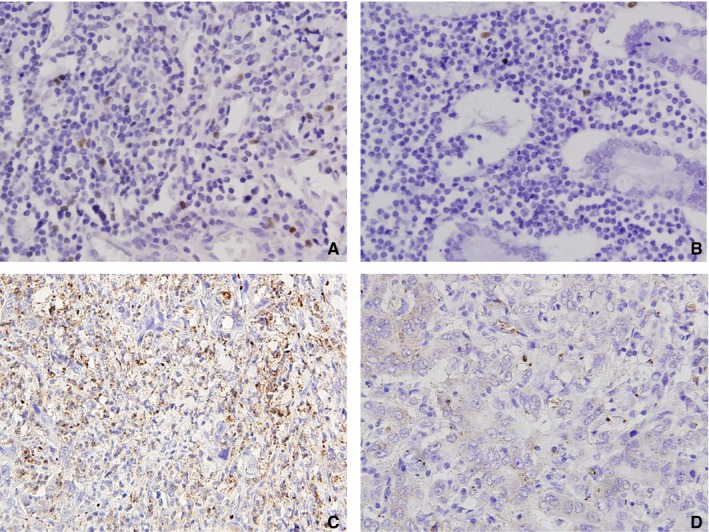
Immunohistochemical staining for Foxp3 + Treg and IDO expression in gastric cancer tissues (×400): (A) Foxp3 + Treg was high (positive cell staining index = 2.9%); (B) Foxp3 + Treg was low (positive cell staining index = 0.2%). (C) IDO expression was positive. (D) IDO expression was negative

Foxp3 was visualized as a brownish, dark yellow stain in lymphocyte nuclei dispersed in the stroma (Figure 3A). IDO in cancer tissues was mainly visualized as a brownish yellow stain in the cytoplasm of tumor cells, although some amount was also seen to be dispersed in the stroma (Figure 3C). Gastric cancer at the T3‐T4 stage exhibited higher levels of tumor‐infiltrating Foxp3 + Tregs (*P* = .007); (Table 2). Patients with larger tumors (>5 cm) and at more advanced T stages (T3 + T4) were more frequently positive for IDO expression (*P* = .044 and *P* = 0027, respectively).

### 3.4 The association between peripheral and tumor‐infiltrating T cells, DCs, IDO expression, and Foxp3 + Treg

**Figure 4:**
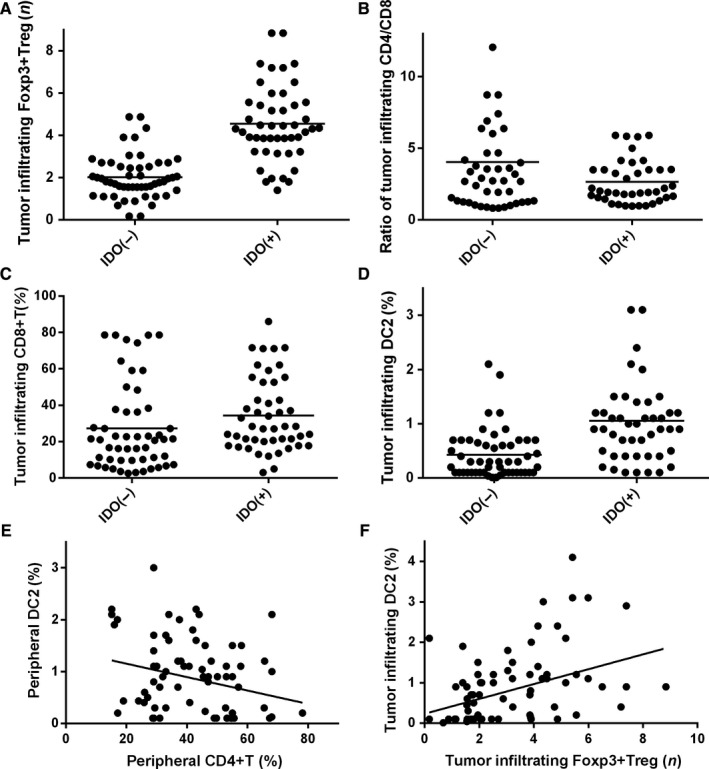
The correlation between peripheral T cell, DCs, and tumor‐infiltrating T cell, DCs, IDO expression, and Foxp3 + Treg. (A) tumor‐infiltrating IDO expression was correlated with Foxp3 + Treg; (B) tumor‐infiltrating IDO expression was correlated with ratio of tumor‐infiltrating DC1/DC2; (C) tumor‐infiltrating IDO expression was correlated with tumor‐infiltrating CD8 + T cells; (D) tumor‐infiltrating IDO expression was correlated with tumor‐infiltrating DC2; (E) peripheral CD4 + T cells were correlated with peripheral DC2 negatively; (F) tumor‐infiltrating Foxp3 + Treg cells were correlated with tumor‐infiltrating DC2 positively

We analyzed the association between peripheral and tumor‐infiltrating T cells, DCs, IDO, and Foxp3 + Tregs in tumor tissues. IDO‐positive patients had higher levels of tumor‐infiltrating Foxp3 + Treg cells (*t* = 8.686; *P* < .001, Figure 4A) and tumor‐infiltrating DC2s (*t* = 4.543, *P* < .001, Figure 4D), but a lower tumor‐infiltrating CD4/CD8 + T cell ratio (*t* = 2.323, *P* = .023, Figure 4B). Peripheral CD4 + T cells were negatively correlated with peripheral DC2s (*r* ^2^ = 0.671; *P* = .007, Figure 4E). Tumor‐infiltrating Foxp3 + Tregs were positively correlated with tumor‐infiltrating DC2s (*r* ^2^ = 0.772; *P* < .001, Figure 4F). Tumor‐infiltrating CD8 + T cell was also correlated with IDO expression via Spearman correlation analysis (*r* ^2^ = 0.441, *P* = .016, Figure 4C).

### 3.5 Prognostic analysis

**Figure 5:**
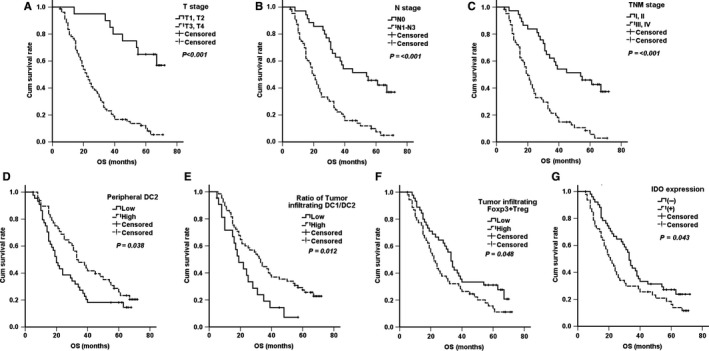
Significant prognostic factors for gastric cancer patients. (A) T stage; (B) N stage; (C) TNM stage; (D) peripheral DC2; (E) Ratio of tumor‐infiltrating DC1/DC2; (F) Tumor‐infiltrating Foxp3 + Treg; (G) Tumor‐infiltrating IDO expression

In the survival analysis, peripheral DC2s (*P* = .038), tumor‐infiltrating DC1/DC2 cell ratios (*P* = .012), Foxp3 + Tregs (*P* = .048), and IDO expression (*P* = .043) were significantly correlated with OS, at T (*P* < .01), N (*P* < .01), and TNM (*P* < .01) stages (Figure 5). Survival rates based on immunological features and clinicopathological features are listed in Table 3. COX regression was used to obtain adjusted hazard ratios for prognosis (Table 4). The T stage and peripheral DC2 cells were shown to be significant risk factors for OS [HR: 0.227 (0.086‐0.601), *P* = .003; HR: 1.978 (1.150‐3.404), *P* = .014].

**Table 3:** 1, 3, and 5 y survival rate of gastric cancer patients according to clinicopathological and immune features

Clinicopathological features	Total	OS				
		1‐YSR	3‐YSR	5‐YSR	*x* ^2^	*P*
Age						
<50	51	80.4	35.5	19.6	0.000	.993
≥50	48	85.1	38.2	27.4		
Sex						
Male	63	83.9	35.5	19.6	0.432	.511
Female	36	80.6	38.9	38.9		
Tumor size						
<5 cm	57	91.2	45.6	27.8	2.894	.089
≥5 cm	42	70.8	24.4	13.7		
Bormann type						
I + II	37	86.1	41.7	24.3	0.378	.534
III + IV	62	80.6	33.9	20.6		
T stage						
T1, T2	20	100	90	65.0	26.986	<.01
T3, T4	79	78.2	23.1	10.1		
N stage						
N0	35	97.1	62.9	45.7	22.233	<.01
N1‐N3	64	74.6	22.2	9.9		
TNM stage						
I, II	37	97.3	62.2	45.9	24.494	<.01
III, IV	62	75.4	21.3	5.6		
Histological grade(‐differentiated)						
Well	28	72.2	32.9	15.8	3.625	.057
Poor	71	96.4	46.4	35.7		
Peripheral CD4 + T cells^a^						
Low	45	80.9	36.2	20.5	0.017	.896
High	54	84.3	37.2	22.7		
Peripheral CD8 + T cells^a^						
Low	49	79.6	49.0	28.0	1.492	.222
High	50	85.7	32.7	1.54		
Ratio of peripheral CD4 + T/CD8 + T cells^a^						
Low	48	76.6	31.9	16.8	1.898	.168
High	51	84.3	43.1	26.5		
Peripheral DC1 cells^a^						
Low	44	87.2	30.8	20.5	0.421	.517
High	54	81.1	41.5	23.8		
Peripheral DC2 cells^a^						
Low	47	77.3	27.3	18.2	4.313	.038
High	52	89.6	47.6	26.0		
Peripheral DC1/DC2 cells^a^						
Low	49	88.9	24.1	18.1	0.004	.949
High	50	78.7	38.3	25.5		
Tumor‐infiltrating CD4 + T cells^a^						
Low	45	82.2	44.4	25.3	1.100	.299
High	54	83.0	32.0	18.7		
Tumor‐infiltrating CD8 + T cells^a^						
Low	48	81.3	41.7	21.7	0.374	.541
High	51	84.0	32.0	21.8		
Ratio of Tumor‐infiltrating CD4 + T/CD8 + T cells^a^						
Low	49	83.7	32.7	26.4	0.083	.773
High	50	81.7	40.8	17.0		
Tumor‐infiltrating DC1 cells^a^						
Low	48	85.7	33.3	11.9	3.529	.060
High	51	79.0	43.2	33.4		
Tumor‐infiltrating DC2 cells^a^						
Low	48	90.5	38.1	19.0	0.007	.935
High	51	75.0	38.7	26.6		
Ratio of Tumor‐infiltrating DC1/DC2 cells^b^						
Low	28	86.2	44.6	27.4	6.295	.012
High	71	71.8	19.1	7.2		
Foxp3 + Treg^a^						
Low	46	88.9	42.2	37.1	3.898	.048
High	53	77.4	32.1	13.4		
IDO						
‐	52	92.2	31.4	27.2	4.086	.043
+	47	72.3	29.8	16.2		

Abbreviation: YSR, Year survival rate.

a Median was set as cutoff value; Low was ≤ Median; High was > Median.

b Upper quartile was set as cutoff value; Low was ≤ upper quartile; High was > upper quartile.

**Table 4:** COX regression for prognostic features of gastric cancer patients

Features	*β*	SE	Wald	*df*	*P*	HR (95% CI)
T stage						
T1, T2	−1.483	0.497	8.919	1	.003	0.227 (0.086‐0.601)
T3, T4						1
N stage						
N0	−0.423	1.083	0.153	1	.696	0.655 (0.078‐5.468)
N1‐N3						1
TNM stage						
I, II	−0.173	1.144	0.023	1	.880	0.841 (0.089‐7.921)
III, IV						1
Peripheral DC2 cells						
Low	0.682	0.277	6.069	1	.014	1.978 (1.150‐3.404)
High						1
Ratio of Tumor‐infiltrating DC1/DC2 cells						
Low	−0.003	0.322	0.000	1	.992	0.997 (0.530‐1.873)
High						1
Foxp3 + Treg						
Low	0.089	0.368	0.059	1	.809	1.093 (0.531‐2.250)
High						1
IDO						
−	−0.114	0.397	0.083	1	.774	0.892 (0.410‐1.941)
+						1

Note

To get adjusted hazard ratios, COX regression was applied for adjusting significant covariate in Kaplan‐Meier prognostic analysis.

Abbreviations: *β*, regression coefficient; SE, standard error; Wald, Wald Chi‐square; *df*, degree of freedom; HR, hazard ratio.

## 4 DISCUSSION

In the tumor microenvironment, the immune system contributes to both tumor elimination and promotion. Due to the multiple factors and processes of immunological modulation mechanisms involved in cancer progression, every patient displayed a different immune phenotype because of the different types of tumor pathology, diseases stage, and basic disease.

Most studies on DCs found that DC‐related signals were required to enable protective immunity rather than immune tolerance. Yet, high DC2 infiltration has been confirmed in many different cancers, such as breast cancer, lung cancer, ovarian cancer, and melanoma. Tumor‐infiltrating DC2s participate in cancer progression as well as inhibition, based on their maturity and gene regulation ability.^23^, ^24^ DC2s are a complex subset of immune cells derived from the bone marrow. DC2s secrete interferons (IFNs) and play a role as antigen‐presenting cells (APC).^23^ Furthermore, peripheral DC2s have been associated with survival in some malignant tumors. For example, advanced stage breast cancer patients had significantly lower peripheral DC2s.^25^ Moreover, prostate cancer patients have shown a marked reduction in circulating DCs.^26^ Liu et al, also reported that peripheral DC2s may be an important prognostic factor for patients with gastric carcinoma at different stages.^1^ In the current study, lower levels of peripheral DC2s were found in more advanced T stage gastric cancers, where patients with lower peripheral DC2 levels had poorer prognoses. In a previous study, it was also found that certain activated DC2s are able to initiate antitumor immunoreactions.^27^ It was verified that tumor‐infiltrating DC2s are mostly immature and predominantly serve an immunosuppressive/tolerogenic purpose.^28^ In our study, patients with N1‐N3 stage gastric cancer had higher tumor‐infiltrating DC2 levels, while patients with T3‐T4 stage gastric cancer showed a lower ratio of tumor‐infiltrating DC1/DC2s. Thus, a low tumor‐infiltrating DC1/DC2 ratio was an important prognostic factor for shorter survival.

IDO exhibits the immunosuppressive function of T‐cell inhibition. Inducing differentiation and maturation of Treg cells is a crucial pathway influencing the effect of IDO. Many studies have demonstrated that increased IDO expression may enable cancer cells to evade immune cytotoxic effects. IDO is the only cytosolic enzyme that catabolizes L‐Trp to L‐Kyn and its downstream metabolites. Normally, it is expressed at a low level.^29^ Reportedly, overexpression of IDO has been confirmed in the lymphatic drainage areas of many malignant cancers, such as stomach carcinomas,^30^ breast neoplasms,^22^ lung tumors,^31^ pancreatic carcinomas, and colorectal tumors,^32^ among others. In the current study, IDO expression was correlated with a larger tumor size, a more advanced T stage, and poorer prognosis.

In our study, density of tumor‐infiltrating Foxp3 + Treg cells was positively correlated with tumor‐infiltrating DC2s. In previous studies, Treg and DC interaction was dependent on serial mechanisms. Onishi et al, showed that these two cell types were dependent on strong LFA‐1‐ICAM‐1 binding by Tregs.^33^ Additionally, IDO, CD39/CD73 expression induced adenosine and CTLA‐4 promoted elimination of the co‐stimulatory molecules, CD80 and CD86, from the membranes of DCs^34^ have all been attributed to Treg‐induced modification of DCs leading to reduced T‐cell responses. Exposure to Tregs may also alter the ability of DCs to activate T cells and modify cytokine profiles.^35^ They release inhibitory cytokine IL‐10, TGF‐*β*, and IL‐35, while reducing IL‐12 production following TLR activation.^36^

The IDO pathway is an important pathway by which DC2 initiated naive CD4 + T cells convert to Tregs. Once induced by TLR‐9, DC2s upregulate the expression of the B‐7 ligands, HLA‐DR antigen, and IDO, all of which promote Treg induction.^37^ It was demonstrated to be an immune‐evading mechanism, and under clinical conditions, it was related to certain nonimmune prognostic advantages in gastric cancer patients.^37^ IDO expression in this study was associated with tumor‐infiltrating Foxp3 + Treg cells and tumor‐infiltrating DC2 cells, but was correlated with a lower ratio of tumor‐infiltrating CD4/CD8 T cells. Tumor‐infiltrating Foxp3 + Treg cells were positively correlated with tumor‐infiltrating DC2s. IDO and Foxp3 + Tregs were also important prognostic factors in our study.

Yet, the precise mechanism(s) underlying these processes remain unexposed. It is well demonstrated that tumor‐infiltrating CD4 + T cells, which are able to recognize cancer associated antigens, are generated to become Th1 cells and stimulate M1‐macrophages via interleukin‐12 or IFN‐γ production. In our study, gastric cancer patients with smaller tumors (<5 cm) had higher CD4 + T and CD8 + T cell levels. Additionally, patients with well‐differentiated gastric carcinoma had higher levels of CD4 + T cells.

Our study had some limitations: (a) The study was based on a relatively small sample obtained via a cohort from a single center; (b) Foxp3 was applied for labeling Tregs. Although it is commonly used, Foxp3 is also found in activated CD4 + T cells; (c) the results as well as the analyses which followed were based solely on clinical data. Therefore, further investigation of underlying mechanisms may be necessary to elucidate the significance of these molecular processes.

## 5 CONCLUSIONS

Numerous factors such as T cells, DCs, Tregs, and IDO are involved in maintaining immune homeostasis and tolerance. All these factors may influence therapeutic options and clinical outcomes. Immunocompetent cells and humoral immune factors, including DC2s, CD4+/CD8 + T cells, Foxp3 + Tregs, and IDO, interact with each other to compose a complex community of tumor immune microenvironment, ultimately affecting tumor progression and survival of gastric cancer.

## CONFLICT OF INTERESTS

The authors have declared that no competing interests exist.

## AUTHOR CONTRIBUTIONS

Fangxuan Li, Jinchao Huang, and Juntian Liu analyzed and interpreted the patient data. Fangxuan Li, Yao Sun, and Jinchao Huang performed the IHC and histological examination of the cancer tissues, Sun Yao and Fangxuan Li were major contributors in writing the manuscript. Wengui Xu and Zhiyong Yuan were contributed to flow cytometry and revised the manuscript. All authors read and approved the final manuscript.

## COMPLIANCE WITH ETHICAL STANDARDS

Ethics approval and consent to participate: This research project was approved by the Ethics Committee of Tianjin Cancer Institute and Hospital. Written consents were obtained from each patient.

## CONSENT FOR PUBLICATION

Written consents were obtained from each patient to publish their pathological images as represent Figures.

## Data Availability

The datasets used during the current study are available from the corresponding author on reasonable request.

## References

[1] Liu W , Zhao J , Li Q , Wang Q , Zhou Y , Tong Z . Gastric cancer patients have elevated plasmacytoid and CD1c(+) dendritic cells in the peripheral blood. Oncol Lett. 2018;15:5087‐5092. 29552142 10.3892/ol.2018.7990 PMC5840537

[2] Soo RA , Chen Z , Teng R , et al. Prognostic significance of immune cells in non‐small cell lung cancer: meta‐analysis. Oncotarget. 2018;9:24801‐24820. 29872507 10.18632/oncotarget.24835 PMC5973851

[3] Veglia F , Gabrilovich DI . Dendritic cells in cancer: the role revisited. Curr Opin Immunol. 2017;45:43‐51. 28192720 10.1016/j.coi.2017.01.002 PMC5449252

[4] Niccolai E , Taddei A , Prisco D , Amedei A . Gastric cancer and the epoch of immunotherapy approaches. World J Gastroenterol. 2015;21:5778‐5793. 26019442 10.3748/wjg.v21.i19.5778 PMC4438012

[5] Chang WJ , Du Y , Zhao X , Ma LY , Cao GW . Inflammation‐related factors predicting prognosis of gastric cancer. World J Gastroenterol. 2014;20:4586‐4596. 24782611 10.3748/wjg.v20.i16.4586 PMC4000495

[6] Tsukayama S , Omura K , Yoshida K , Tanaka Y , Watanabe G . Prognostic value of CD83‐positive mature dendritic cells and their relation to vascular endothelial growth factor in advanced human gastric cancer. Oncol Rep. 2005;14:369‐375. 16012717

[7] Kashimura S , Saze Z , Terashima M , et al. CD83+ dendritic cells and Foxp3+ regulatory T cells in primary lesions and regional lymph nodes are inversely correlated with prognosis of gastric cancer. Gastric Cancer. 2012;15(2):144‐153. 22083420 10.1007/s10120-011-0090-9

[8] Rissoan MC , Soumelis V , Kadowaki N , et al. Reciprocal control of T helper cell and dendritic cell differentiation. Science. 1999;283:1183‐1186. 10024247 10.1126/science.283.5405.1183

[9] Zou W . Regulatory T cells, tumour immunity and immunotherapy. Nat Rev Immunol. 2006;6:295‐307. 16557261 10.1038/nri1806

[10] Safinia N , Leech J , Hernandez‐Fuentes M , Lechler R , Lombardi G . Promoting transplantation tolerance; adoptive regulatory T cell therapy. Clin Exp Immunol. 2013;172:158‐168. 23574313 10.1111/cei.12052 PMC3628319

[11] Fridman WH , Pages F , Sautes‐Fridman C , Galon J . The immune contexture in human tumours: impact on clinical outcome. Nat Rev Cancer. 2012;12:298‐306. 22419253 10.1038/nrc3245

[12] Badoual C , Hans S , Rodriguez J , et al. Prognostic value of tumor‐infiltrating CD4+ T‐cell subpopulations in head and neck cancers. Clin Cancer Res. 2006;12:465‐472. 16428488 10.1158/1078-0432.CCR-05-1886

[13] Marshall EA , Ng KW , Kung S , et al. Emerging roles of T helper 17 and regulatory T cells in lung cancer progression and metastasis. Mol Cancer. 2016;15:67. 27784305 10.1186/s12943-016-0551-1 PMC5082389

[14] Thackray SJ , Mowat CG , Chapman SK . Exploring the mechanism of tryptophan 2,3‐dioxygenase. Biochem Soc Trans. 2008;36:1120‐1123. 19021508 10.1042/BST0361120 PMC2652831

[15] Maleki VS , Rytelewski M , Figueredo R , et al. Indoleamine 2,3‐dioxygenase mediates immune‐independent human tumor cell resistance to olaparib, gamma radiation, and cisplatin. Oncotarget. 2014;5:2778‐2791. 24784564 10.18632/oncotarget.1916 PMC4058044

[16] Munn DH , Mellor AL . Indoleamine 2,3‐dioxygenase and tumor‐induced tolerance. J Clin Invest. 2007;117:1147‐1154. 17476344 10.1172/JCI31178 PMC1857253

[17] Siegel RL , Miller KD , Jemal A . Cancer statistics, 2018. CA Cancer J Clin. 2018;68:7‐30. 29313949 10.3322/caac.21442

[18] Ying L , Yan F , Meng Q , et al. PD‐L1 expression is a prognostic factor in subgroups of gastric cancer patients stratified according to their levels of CD8 and FOXP3 immune markers. Oncoimmunology. 2018;7:e1433520. 29872566 10.1080/2162402X.2018.1433520 PMC5980489

[19] Yuan X‐L , Chen L , Li M‐X , et al. Elevated expression of Foxp3 in tumor‐infiltrating Treg cells suppresses T‐cell proliferation and contributes to gastric cancer progression in a COX‐2‐dependent manner. Clin Immunol. 2010;134:277‐288. 19900843 10.1016/j.clim.2009.10.005

[20] Zhang R , Liu H , Li F , Li H , Yu J , Ren X . The correlation between the subsets of tumor infiltrating memory T cells and the expression of indoleamine 2,3‐dioxygenase in gastric cancer. Dig Dis Sci. 2013;58:3494‐3502. 23979437 10.1007/s10620-013-2837-0

[21] Li F , Huang J , Li S , et al. The subsets of dendritic cells and memory T cells correspond to indoleamine 2,3‐dioxygenase in stomach tumor microenvironment. Tumour Biol. 2014;35:8691‐8698. 24870595 10.1007/s13277-014-2126-3

[22] Yu J , Sun J , Wang SE , et al. Upregulated expression of indoleamine 2, 3‐dioxygenase in primary breast cancer correlates with increase of infiltrated regulatory T cells in situ and lymph node metastasis. Clin Dev Immunol. 2011;2011:469135. 22110525 10.1155/2011/469135 PMC3202140

[23] Swiecki M , Colonna M . The multifaceted biology of plasmacytoid dendritic cells. Nat Rev Immunol. 2015;15:471‐485. 26160613 10.1038/nri3865 PMC4808588

[24] Lombardi VC , Khaiboullina SF , Rizvanov AA . Plasmacytoid dendritic cells, a role in neoplastic prevention and progression. Eur J Clin Invest. 2015;45(Suppl 1):1‐8. 10.1111/eci.12363 25524580

[25] Kini BJ , Gueckel B , Pawelec G . Prognostic impact of high levels of circulating plasmacytoid dendritic cells in breast cancer. J Transl Med. 2016;14:151. 27234566 10.1186/s12967-016-0905-x PMC4884426

[26] Sciarra A , Lichtner M , Autran GA , et al. Characterization of circulating blood dendritic cell subsets DC123+ (lymphoid) and DC11C+ (myeloid) in prostate adenocarcinoma patients. Prostate. 2007;67:1‐7. 17075798 10.1002/pros.20431

[27] Tel J , Smits EL , Anguille S , Joshi RN , Figdor CG , de Vries IJ . Human plasmacytoid dendritic cells are equipped with antigen‐presenting and tumoricidal capacities. Blood. 2012;120:3936‐3944. 22966165 10.1182/blood-2012-06-435941

[28] Demoulin S , Herfs M , Delvenne P , Hubert P . Tumor microenvironment converts plasmacytoid dendritic cells into immunosuppressive/tolerogenic cells: insight into the molecular mechanisms. J Leukoc Biol. 2013;93:343‐352. 23136258 10.1189/jlb.0812397

[29] Takikawa O . Biochemical and medical aspects of the indoleamine 2,3‐dioxygenase‐initiated L‐tryptophan metabolism. Biochem Biophys Res Commun. 2005;338:12‐19. 16176799 10.1016/j.bbrc.2005.09.032

[30] Zhang R , Li H , Yu J , et al. Immunoactivative role of indoleamine 2,3dioxygenase in gastric cancer cells in vitro. Mol Med Report. 2011;4:169‐173. 10.3892/mmr.2010.398 21461581

[31] Astigiano S , Morandi B , Costa R , et al. Eosinophil granulocytes account for indoleamine 2,3‐dioxygenase‐mediated immune escape in human non‐small cell lung cancer. Neoplasia. 2005;7:390‐396. 15967116 10.1593/neo.04658 PMC1501151

[32] Brandacher G , Perathoner A , Ladurner R , et al. Prognostic value of indoleamine 2,3‐dioxygenase expression in colorectal cancer: effect on tumor‐infiltrating T cells. Clin Cancer Res. 2006;12:1144‐1151. 16489067 10.1158/1078-0432.CCR-05-1966

[33] Onishi Y , Fehervari Z , Yamaguchi T , Sakaguchi S . Foxp3+ natural regulatory T cells preferentially form aggregates on dendritic cells in vitro and actively inhibit their maturation. Proc Natl Acad Sci USA. 2008;105:10113‐10118. 18635688 10.1073/pnas.0711106105 PMC2481354

[34] Chen X , Du Y , Hu Q , Huang Z . Tumor‐derived CD4+CD25+regulatory T cells inhibit dendritic cells function by CTLA‐4. Pathol Res Pract. 2017;213:245‐249. 28214198 10.1016/j.prp.2016.12.008

[35] Ring S , Karakhanova S , Johnson T , Enk AH , Mahnke K . Gap junctions between regulatory T cells and dendritic cells prevent sensitization of CD8(+) T cells. J Allergy Clin Immunol. 2010;125:237‐46.e1‐7. 20109751 10.1016/j.jaci.2009.10.025

[36] Mavin E , Nicholson L , Rafez AS , Gao F , Dickinson A , Wang XN . Human regulatory T cells mediate transcriptional modulation of dendritic cell function. J Immunol. 2017;198:138‐146. 27895173 10.4049/jimmunol.1502487

[37] Munn DH , Mellor AL . IDO in the tumor microenvironment: inflammation, counter-regulation, and tolerance. Trends Immunol. 2016;37(3):193‐207. 26839260 10.1016/j.it.2016.01.002 PMC4916957

